# Nafion in Biomedicine and Healthcare

**DOI:** 10.3390/polym17152054

**Published:** 2025-07-28

**Authors:** Antonios Kelarakis

**Affiliations:** UCLan Research Centre for Smart Materials, School of Pharmacy and Biomedical Sciences, University of Central Lancashire, Preston PR1 2HE, UK; akelarakis@uclan.ac.uk

**Keywords:** Nafion, healthcare, biomedicine, biosensors, controlled drug delivery, antimicrobial, lab-on-a-chip, proton exchange membrane, electrochemical characteristics, bioelectronic system, ion exchange capacity

## Abstract

Nafion has long been recognized as the gold standard for proton exchange membranes, due to its exceptional ion exchange capacity and its advanced performance in chemically aggressive environments. In recent years, a growing body of evidence has demonstrated that Nafion is equally well-suited in complex biological conditions owing to its structural robustness, responsive functionality and intrinsic biocompatibility. These characteristics have enabled its transition into the biomedical and healthcare sectors, where it is currently being explored for a diverse and expanding range of applications. To that end, Nafion has been systematically investigated as a key component in bioelectronic systems for energy harvest, sensors, wearable electronics, tissue engineering, lab-on-a-chip platforms, implants, controlled drug delivery systems and antimicrobial surface coatings. This review examines the distinctive structural and electrochemical characteristics that underpin Nafion’s performance in these biomedical contexts, provides an overview of recent advancements, emphasizes critical performance metrics and highlights the material’s growing potential to shape the future of biomedical technology.

## 1. Introduction

By virtue of its chemical design, a Teflon-like polytetrafluoroethylene (PTFE) backbone bearing sulfonic acid pendant groups, Nafion shows a unique combination of highly desirable characteristics, including superior mechanical robustness, remarkable chemical and thermal stability and advanced proton conductivity. This structural duality leads to microphase separation, where hydrophilic domains formed by sulfonic acid clusters create well-defined, continuous water channels that enable efficient proton conduction, while the hydrophobic PTFE backbone provides mechanical integrity and resistance to harsh chemical environments [1,2]. The high density of these ionizable sites makes Nafion a benchmark material for proton exchange membranes (PEMs) in electrochemical settings. In particular, Nafion is widely used in Proton Exchange Membrane Fuel Cells (PEMFCs) [3,4], Proton Exchange Membrane Water Electrolysis (PEMWE) [5] and redox flow batteries, where the incorporation of functional additives or structural modifications is often employed to further improve the stability and durability of the membranes [6]. Moreover, due to its amphiphilic nature, Nafion can interact with a range of polymeric compounds and nanomaterials giving rise to supramolecular assemblies [7,8,9] and functional nanocomposites [10,11].

Nafion’s excellent processability allows for its fabrication into films, membranes and coatings via scalable methods such as solution casting, spin coating, dip coating and spray deposition. It can be uniformly applied to substrates including ceramics, metals and polymers, expanding its use in areas like humidity and gas sensors [12,13], environmental monitoring [14,15] and detoxification [16]. Nafion-based actuators exploit its ionic electroactive properties to mimic the voltage-induced motion of biological muscles, enabling their application in soft robotics [17,18].

Furthermore, Nafion is non-toxic, it does not elicit an immune response, and it remains stable in physiological environments, making it particularly attractive for an expanding range of biomedical and health-related applications [19,20]. For example, Nafion-based membranes are widely employed in biosensors due to their ability to selectively permit the passage of target ions or molecules [21]. In addition, Nafion has been integrated into microfluidic and lab-on-a-chip devices, making it ideal for diagnostics, biochemical assays, high-throughput screening and portable analytical platforms [22]. Beyond sensing, Nafion is also being explored in advanced bioelectronics for energy harvest [23]. In drug delivery, Nafion is used in stimuli-responsive platforms where the release of therapeutic agents can be modulated by environmental factors such as pH [24].

This review examines how Nafion’s remarkable structural and electrochemical characteristics underpin these innovations, addressing recent advances, performance metrics and future prospects for its use in next-generation biomedical engineering. To the best of our knowledge, no comprehensive review currently exists that presents these emerging developments in the healthcare domain. In that sense, this review seeks to fill that void by compiling the state-of-the-art advancements in Nafion-based technologies tailored to biological and clinical applications.

## 2. Discussion

### 2.1. Bioelectronic Systems for Energy Harvest

Currently, most bioelectronic devices (such as pacemakers, cochlear implants, deep brain stimulators, spinal cord stimulators, insulin pumps, electroencephalography devices, electrocardiography monitors, etc.) depend on rechargeable batteries or wireless systems, which pose challenges in reliability and user convenience. Nafion-based energy harvesting systems offer a promising solution by enabling the development of autonomous, self-sustaining implantable devices that eliminate the need for external power sources, enhancing device longevity and patient comfort.

**Figure 1:**
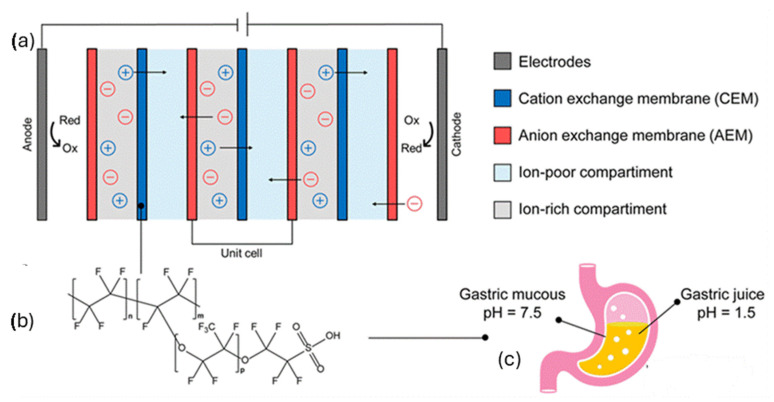
(**a**) Energy generation via reverse electrodialysis that relies on the use of Nafion (**b**) as the cation exchange membrane (CEM) and exploits the ionic gradient between the gastric mucous and the gastric juice (**c**). Adopted from reference [25].

A recent report highlights the potential of Nafion membranes to harvest energy in vivo capitalizing on the six-order magnitude proton concentration gradient between the highly acidic gastric juice (pH ~1.5) and the near-neutral gastric mucosa (pH ~7.5) [25]. Figure 1 displays the energy conversion mechanism that is based on reverse electrodialysis (RED), wherein a series of ion-selective membranes, alternating between cation exchange membranes (CEMs) such as Nafion and anion exchange membranes (AEMs), are exposed to ion-rich and ion-poor compartments. As protons migrate through the membranes, a voltage is created across each membrane pair, in a manner that the overall potential increases with the number of stacked cells. The prototype developed in this study shows that a single RED unit can generate a potential difference of 134 mV and a power density of 188 mW/m^2^, demonstrating the feasibility of using gastric pH gradients to power biomedical electronics.

Another study demonstrated a metabolic fuel cell that connects to the bloodstream and utilizes an anode comprising nanoparticles (NPs) of CuO/carbon nanotube (CNT) composite where under hyperglycaemic conditions glucose is converted to gluconate with concomitant release of electrons that produce an electric signal and protons [26]. The protons travel to cathode, composed of Nafion-coated carbon black (CB)-containing platinum NPs (Pt-CB/Nafion), where they are catalytically recombined with oxygen to form water. This device lowers the glucose levels in blood (glucose is consumed in anode) and utilizes the harnessed energy to trigger vesicular insulin release via optostimulation of Opto-β cells or electrostimulation of Electro-β cells. Once glucose levels return to physiological norms, the devise automatically ceases operation, essentially functioning as a closed-loop feedback system for glucose monitoring and regulation. In experimental models of type 1 diabetes, this metabolic fuel cell successfully restores glucose homeostasis autonomously, without the need for external intervention.

In addition, the successful fabrication of Nafion-based piezoionic (PIOG) and triboelectric (TEG) energy harvesting devices was demonstrated, generating approximately 450 mV and 1.9 V, respectively, per finger or hand tap [27]. The PIOG device was made by placing a tin-doped indium oxide (ITO) electrode on the Nafion film and sealing it with Kapton tape to prevent movement and short circuits. The TEG device was assembled by placing a PET/ITO electrode film and Nafion-coated substrate on a cardboard support, with a sponge spacer creating a gap between the Nafion and the top electrode. Both devices had connecting wires attached to the aluminum and ITO electrodes. In the PIOG device, mechanical tapping deforms the Nafion film, resulting in ion redistribution that generates a voltage across the electrodes, while releasing the pressure reverses this process, in essence resulting in an alternating electric current. In the TEG device, voltage generation occurs through contact and separation between an ITO electrode and a Nafion film. Upon mechanical force application, charge transfer occurs as ITO becomes positively charged and Nafion negatively charged, while releasing the force induces an electric potential through electrostatic induction.

### 2.2. Biosensing

Nafion’s dynamic presence in the field of electrochemical biosensors can be attributed to its exceptional ionic conductivity, chemical stability and biocompatibility. By design, its structure allows selective cation transport while repelling anions, reducing interference and enhancing selectivity, which is often quantified in ion-conducting membranes as the ratio of proton conductivity to the conductivity of other ionic species. Nafion also acts as a stable immobilization matrix for biomolecules such as enzymes, antibodies and nucleic acids, preserving their activity and improving sensor stability [21]. Additionally, Nafion layers can amplify electrochemical signals by promoting the accumulation of reaction products at the electrode surface, while its anti-biofouling properties help maintain sensor performance in complex biological environments [28].

A study focuses on the development of a flexible, regenerative aptameric field-effect transistor biosensor that effectively detects cytokine storm biomarkers in human biofluids [29]. By incorporating a graphene–Nafion composite film, the biosensor minimizes nonspecific adsorption with a limit of detection (LOD) as low as 740 fM in undiluted human sweat for biomarkers such as interferon-gamma, a key indicator of inflammation and infection. Importantly, the device maintains structural integrity and reliable performance up to 80 regenerative and 100 mechanical crumpling cycles, confirming its potential for early intervention and continuous monitoring in acute and chronic inflammatory conditions.

Another study highlights the integration of Nafion membranes with nanoporous gold electrodes to enhance electrochemical aptamer-based biosensing for the detection of the cationic chemotherapeutic drug doxorubicin [30]. The resulting biosensor demonstrates excellent sensitivity, selectivity and long-term stability, showing great promise for in vivo and long-term biosensing applications.

**Figure 2:**
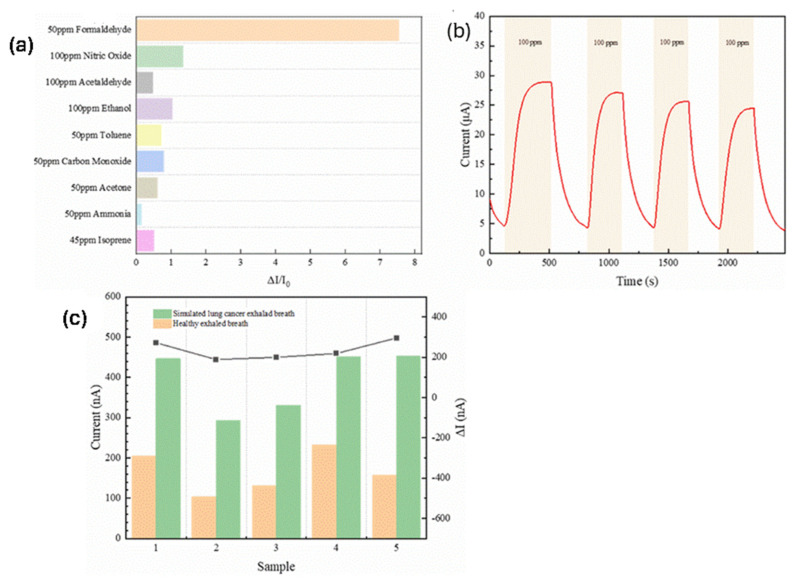
(**a**) Selectivity and (**b**) cycle stability of Nafion-based HCHO sensor. (**c**) Sensor’s response to healthy individuals and simulated lung cancer patients. Adopted from reference [31].

Moreover, a high-performance electrochemical sensor for detecting formaldehyde (HCHO), a key biomarker for lung cancer screening, has been disclosed. The sensor is fabricated via a two-step drop-coating method on a flexible screen-printed electrode, using a 60% Pt/C-Nafion catalyst as the sensing layer and Nafion as the solid-state electrolyte [31]. Operating at 0.5 V, the device achieves high sensitivity (0.197 μA/ppm), a wide detection range within 500 ppb–100 ppm and an impressively low LOD of 2.5 ppb at room temperature. As shown in Figure 2a the sensor demonstrates high selectivity for HCHO given that its response to HCHO appears at least five times greater than nitric oxide, acetaldehyde, ethanol, acetone, toluene, carbon monoxide, ammonia, isoprene. Figure 2b indicates that the sensor’s response when subjected to repeated exposure to 100 ppm HCHO and synthetic air remains relatively stable over multiple cycles. Additionally, tests at ultra-low concentrations (50–400 ppb) show clear differentiation between breath profiles of healthy individuals and simulated lung cancer patients, as shown in Figure 2c.

Nitric oxide is a versatile signaling molecule involved in vascular regulation, neural transmission and disease pathology, but due to its high reactivity and transient nature its accurate detection in biological systems presents challenges. A Pt sensor coated with Nafion multilayers shows a LOD close to 5 nM nitric oxide that is not affected by the interfering molecules such as dopamine, ascorbic acid, nitrite, hydrogen peroxide and oxygen [32]. In vivo experiments involving systemic administration of nitric oxide and L-arginine in freely moving rats indicated that the sensor can provide reliable nitric oxide readings. In addition, the Pt-Nafion sensor was successfully used to monitor the release profile of nitric oxide liberated from sodium nitroprusside, a nitric oxide-donor drug used clinically to treat severe hypertension, in the striatum of anesthetized mice using local administrations as well as freely moving mice using systemic administrations [33].

A D-serine biosensor (D-serine is an amino acid involved in nerve signaling and synaptic plasticity) was developed by immobilizing the enzyme D-amino acid oxidase on a platinum-iridium (Pt-Ir) disk electrode coated with poly-ortho-phenylenediamine and Nafion [34]. The biosensor shows a response time as low as 0.7 s with LOD close to 20 nM, while it is not affected by ascorbic acid, a common interfering substance in the brain. Implantation of the electrode biosensor followed by D-serine microinjection into the rat brain striatal extracellular fluid confirmed its ability to detect D-serine in vivo.

Poly(3,4-ethylenedioxythiophene) (PEDOT):Nafion-coated electrodes, electrodeposited on microelectrode arrays for neural recording, have demonstrated significantly reduced polarization during electrical stimulation and achieved nearly 80% higher charge injection compared to PEDOT:PSS (the current standard in neural interfaces), highlighting their potential for more efficient and safer neural applications [35]. A follow-up study demonstrated that waterborne PEDOT:Nafion coatings are not cytotoxic against rat fibroblasts [36].

Another report presents the development of highly selective dopamine biosensors using CNT field-effect transistors (CNT-FETs) with floating electrodes functionalized with 2,2′-azino-bis(3-ethylbenzothiazoline-6-sulfonic acid) radicals embedded in Nafion films [37]. This type of biosensor achieves a low LOD of 10 nM dopamine, even in the presence of interfering neurotransmitters such as glutamate and acetylcholine. Moreover, the sensors effectively monitor real-time dopamine release from PC12 cells stimulated with high potassium concentrations and were successfully used to assess the effects of the antipsychotic drug pimozide in a dose-dependent manner.

**Figure 3:**
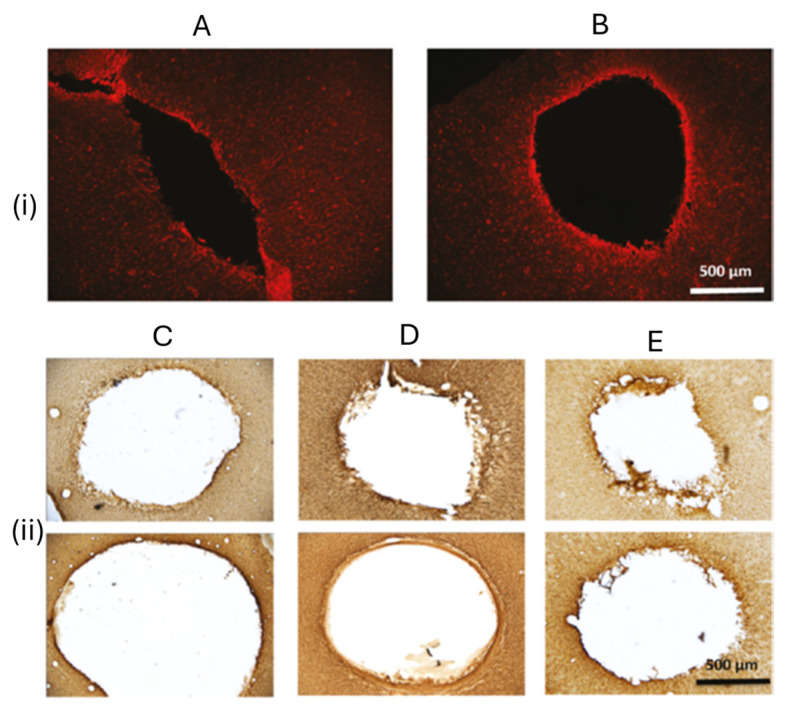
Glial fibrillary acidic protein (GFAP) immunoreactivity in cortical astroglia following reference electrode removal. (**i**) Fluorescence microscopy images depict the tissue surrounding the implantation site of (**A**) a bare Ag/AgCl reference electrode and (**B**) a Nafion-coated electrode after 14 days. (**ii**) Bright-field images show GFAP immunoreactivity around representative lesion sites for bare (top) and Nafion-coated (bottom) electrodes at 7 days (**C**), 14 days (**D**), and 28 days (**E**) post-implantation, demonstrating differences in glial activation over time. Adopted from reference [38].

Chronically implanted Ag/AgCl reference electrodes often exhibit a significant potential shift in the anodic and cathodic peaks of dopamine cyclic voltammograms, compromising the accuracy of long-term neurochemical monitoring. However, this electrochemical drift is substantially reduced when the electrodes are coated with Nafion, which provides stable and reliable readings for up to 28 days [38]. Although extensive glial encapsulation was observed around both coated and uncoated electrodes, the tissue surrounding the bare implants appeared rough and disrupted, indicative of stronger cellular adhesion. The lesion border surrounding the bare electrodes appears rough, suggesting cellular disruption or tearing upon electrode removal. In contrast, the lesion border around Nafion-coated electrodes appears smooth, indicating reduced cellular disturbance. This distinction is also evident in the bright field images in Figure 3(ii), which compares bare (top) and Nafion-coated (bottom) electrodes at 7 (C), 14 (D), and 28 (E) days post-implantation.

A highly sensitive electrochemical sensor was developed using a carbon paste electrode modified with Au NPs and Nafion. This sensor effectively detects catecholamine neurotransmitters, namely dopamine, epinephrine, L-norepinephrine, L-3,4-dihydroxyphenylalanine and serotonin, even in the presence of common interfering substances such as uric acid and ascorbic acid. The sensor demonstrated excellent peak separation in simultaneous measurements of dopamine with serotonin and L-3,4-dihydroxyphenylalanine with acetaminophen (APAP), enabling selective detection with low LOD [39].

**Figure 4:**
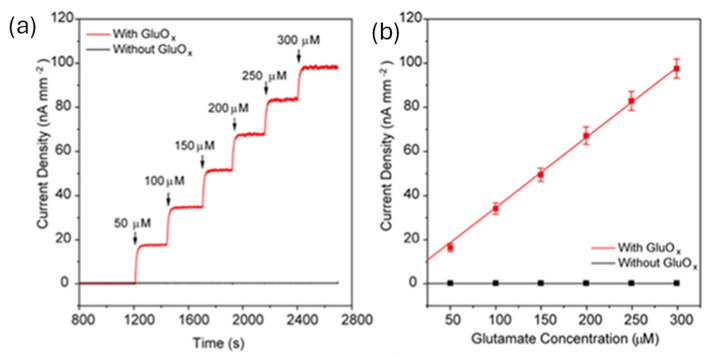
(**a**) Representative amperometric responses of the Nafion-coated NdNiO glutamate biosensor recorded at an applied potential of 0.6 V vs. Ag/AgCl in 0.01 M Phosphate-buffered saline (pH = 7.4), both in the presence and absence of Glutamate Oxidase (GluOx). (**b**) The corresponding calibration plots demonstrate a linear relationship between current density and glutamate concentration. Adopted from reference [40].

A glutamate sensor (glutamate is a major excitatory neurotransmitter critical for synaptic transmission and neurological disorder) based on a perovskite nickelate–Nafion heterostructure was developed, wherein glutamate oxidase (GluOx), an enzyme that metabolizes glutamate and generates H_2_O_2_, was immobilized on Nafion-coated NdNiO_3_ (NNO) films. This sensor achieved an LOD of 16 nM and demonstrated efficacy both ex vivo, in electrically stimulated brain slices, and in vivo, in the primary visual cortex of awake mice exposed to visual stimuli [40]. As shown in Figure 4a, the Nafion–GluOx-modified perovskite nickelate film exhibited a strong electrocatalytic response to incremental additions of glutamate, with each 50 μM dose producing a distinct rise in current density (~18 nA/mm^2^ per addition). This response indicates efficient enzymatic oxidation of glutamate and the subsequent Faradaic reaction (H_2_O_2_ → O_2_ + 2H^+^ + 2e^−^), while control films lacking the Nafion–GluOx functionalization showed no significant current response. The calibration curve shown in Figure 4b demonstrates a linear response with a sensitivity of 0.327 nA μM^−1^·mm^−2^.

### 2.3. Wearable Electronics and Electronic Textiles

Wearable electronics and electronic textiles (e-textiles) represent cutting-edge technologies that seamlessly incorporate sensors, energy storage devices and conductive fibers into garments or accessories. These systems enable continuous, non-invasive tracking of physiological parameters like heart rate, temperature and respiration, thus playing an increasingly important role in healthcare and fitness monitoring. A key element in their design is the integration of electronically conductive fibers or yarns, which are essential for reliable signal transmission, sensing and actuation, while also being able to withstand washing and mechanical stresses such as bending and stretching.

**Figure 5:**
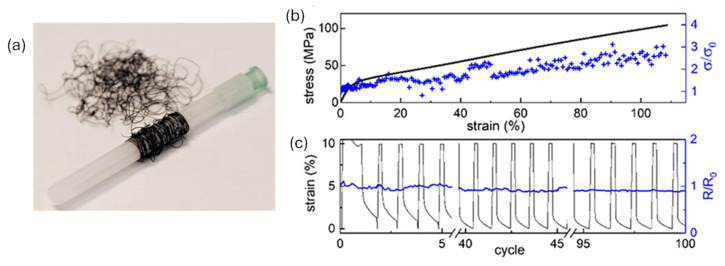
(**a**) Photograph of melt-spun PEDOT:Nafion fibers, wrapped around a 5 mm wide syringe cover. The evolution of electrical conductivity in PEDOT:Nafion fibers was evaluated during uniaxial stretching up to the point of mechanical failure (**b**) and during cyclic deformation within the elastic regime (**c**). Adopted from reference [41].

Currently, Nafion plays a dual role in advancing this field. First, its ion-conducting properties support autonomous energy harvesting mechanisms, including TEG generators, metabolic fuel cells, and RED systems as discussed above, ultimately enabling wearable systems to operate independently of external power sources. Secondly, Nafion serves as a structural template for producing stretchable conductive fibers. A recent study demonstrated a melt-processing technique to fabricate conductive polymer structures by polymerizing PEDOT within a Nafion matrix template [41]. The mechanical characterization of the PEDOT:Nafion fibers (shown in Figure 5a) revealed that the resulting fibers are highly stretchable, exhibiting a Young’s modulus of approximately 620 ± 100 MPa and an elongation at break exceeding 100%. In addition, the fibers demonstrate excellent fatigue resistance, withstanding at least 100 cycles of strain and release at 10% maximum strain without any detectable loss in electrical conductivity (Figure 5b). The resulting fibers show excellent flexibility, maintain electronic conductivity (~3 s/cm) even under 100% strain (Figure 5c), and are compatible with organic electrochemical transistors.

**Figure 6:**
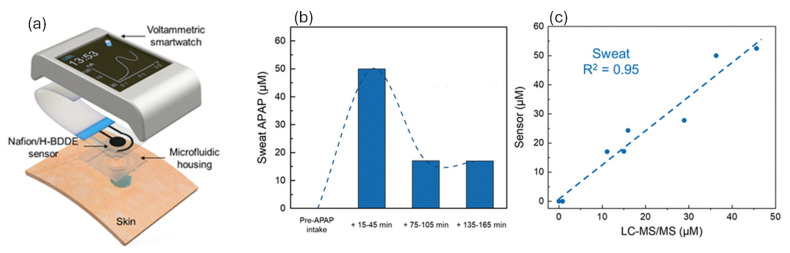
(**a**) The components of a voltammetric smartwatch, which can render APAP readouts. Nafion/H-BDDE-enabled ex situ APAP quantification in sweat. (**b**) Sensor-measured APAP concentration in the sweat of a human subject, collected before and at intermittent time points after the oral administration of a medication containing 650 mg APAP. (**c**) Sensor-measured APAP concentrations in saliva sweat samples versus the corresponding LC-MS/MS readouts. Adopted from Reference [42].

In addition, a sensing platform based on a Nafion-coated, hydrogen-terminated, boron-doped diamond electrode (Nafion/H-BDDE) was embedded within a custom smartwatch and was specifically engineered to track the temporal concentration profile of acetaminophen (APAP) in biofluids such as saliva and sweat (Figure 6a) [42]. The system detected a swift rise in sweat APAP levels following the intake of a 650 mg APAP-based medication, which was subsequently followed by a gradual decline, as illustrated in Figure 6b. The sensor-generated concentration data were validated against laboratory-based liquid chromatography–tandem mass spectrometry (LC-MS/MS), showing strong correlation (R^2^ = 0.95), confirming the sensor’s accuracy in non-invasive, real-time drug monitoring via sweat analysis (Figure 6c).

### 2.4. Tissue Engineering and Regenerative Medicine

Porous chitosan membrane (PCSM) coatings were deposited onto Nafion and polytetrafluoroethylene (PTFE) substrates to form PCSM-Nafion and PCSM-PTFE composites, respectively, and were subcutaneously implanted into rats [43]. Histological assessment at 35, 65, and 100 days post-implantation indicated that PCSM-Nafion demonstrated superior performance, with significantly lower collagen accumulation and a continuous increase in vascularization over time, while PCSM-PTFE composites exhibited higher collagen density and a decline in angiogenesis within the membrane at later stages.

It has been reported that although HEp-2 human cells adhered and reached confluence on both Nafion and poly(vinylidene fluoride-co-hexafluoropropylene) (PVFHFP) films within 7–8 days, cells on Nafion appeared more viable and stable [20]. Subcutaneous implantation studies in mice confirmed Nafion’s superior biocompatibility, showing only a mild acute inflammatory response without chronic inflammation or tissue damage, while PVFHFP caused a stronger and more prolonged reaction.

Another study demonstrated that Ti surfaces coated with Nafion stabilized anatase TiO_2_ NPs showed good adhesion and spreading of human osteoblastic sarcoma cells [44], further highlighting the potential of Nafion-mediated nanocoatings in improving biomaterial interfaces for orthopedic applications.

In vitro studies demonstrated that Nafion functionalized CNTs (Nafion/CNT) when administered to rats that experienced acute spinal cord injury can enhance neurite outgrowth, promoting axonal regeneration within the lesion site, ultimately improving recovery without exacerbating reactive gliosis and minimizing the risks of paraplegia or quadriplegia [45]. Importantly, treated animals exhibited modest improvements in hind limb locomotor function, with no signs of increased pain sensitivity (hyperalgesia).

Micropatterned Nafion films on Si wafers, fabricated using an aryl mold, have been shown to promote cell adhesion and growth. This effect is attributed to the guidance provided by the morphological features of the Nafion patterns coupled with the contrast in stiffness between the Nafion film and the Si substrate [46].

### 2.5. Lab-on-a-Chip Microfluid Devices

Electrokinetic concentration devices enable preconcentration of analytes before detection, significantly enhancing the sensitivity and accuracy of analytical techniques. One of the most effective methods for such preconcentration is electrokinetic trapping, which relies on the use of charge-selective membranes such as Νafion. In electrokinetic trapping, Nafion’s ion permselectivity creates concentration polarization zones when an electric field is applied, ultimately leading to ion depletion on one side and enrichment on the other. This gradient enables the accumulation of charged biomolecules (like proteins or DNA) near the membrane, significantly increasing their local concentration and allowing for more accurate and sensitive downstream detection.

**Figure 7:**
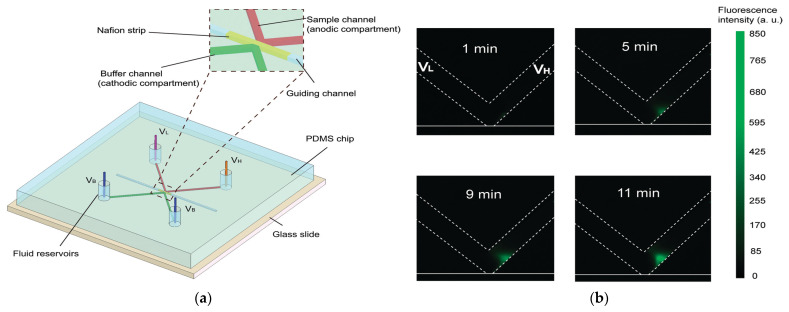
(**a**) Schematic description of a protein preconcentration chip integrating a Nafion strip demonstrating the buffer and the sample channels and the corresponding voltages (V_b_ and V_L_/V_H_, respectively). The magnified view highlights the guiding channel designed for precise Nafion strip placement. (**b**) Fluorescence images showing preconcentration of 6 nM AF-BSA at 1, 5, 9, and 11 min after applying 10 V (V_H_ = 15 V, V_L_ = 5 V); buffer reservoirs held at 0 V. Adopted from Reference [47].

Figure 7 presents a simple microfluidic device for protein preconcentration based on electrokinetic trapping, using a Nafion strip integrated into a Polydimethylsiloxane (PDMS) structure and sealed with a glass substrate [47]. The device enables rapid, leak-tight, and disposable fabrication and can concentrate negatively charged fluorescent proteins near the anodic side of the Nafion strip by up to 10^4^-fold within minutes. As shown in Figure 7a the buffer channel (cathodic compartment) has its fluidic reservoirs grounded (V_B_ = 0 V), while the sample channel (anodic compartment) is connected to two different positive voltages, V_H_ and V_L_, at its respective reservoirs. Figure 7b presents fluorescence images captured at 1, 5, 9, and 11 min during preconcentration of a 6 nM sample under a 10 V potential (V_H_ = 15 V, V_L_ = 5 V) applied across the anodic compartment. It can be seen that a distinct fluorescence spot appears in the lower right region of the sample channel, intensifying over time and the preconcentrated plug remains confined in space and does not extend across the full channel width.

In addition, a microfluidic sample preconcentration chip combining a PDMS microchannel with a graphene oxide (GO)–Nafion nanomembrane was developed and evaluated using fluorescein. It was found that incorporating GO into Nafion significantly enhanced the enrichment efficiency, achieving a 60-fold preconcentration compared to 40-fold for pure Nafion [48]. This improvement is attributed to the dissociated carboxylate groups in GO, which increase cation selectivity within the membrane’s nanopores. However, this enhancement comes with a trade-off in speed, requiring 30 min for the preconcentration process compared to only 6 min and 36 s for the pure Nafion membrane.

Protein crystallization plays a pivotal role in healthcare by enabling the determination of protein structures that is essential in understanding function and advancing drug development. To that end, a cost-effective, high-throughput microfluidic device incorporating a Nafion membrane has been developed to enhance both crystallization and derivatization processes. This system facilitates parallel screening of up to 75 conditions with precise control over water and ion transport [49]. Its efficacy was demonstrated using lysozyme and Hg^2+^, yielding structurally stable crystals with excellent X-ray diffraction quality, thus improving protein structure resolution and contributing to structure-informed drug discovery.

Another study demonstrated that a Nafion-coated Au electrode was effective in preventing irreversible DNA adsorption and degradation, allowing efficient DNA capture and release [50]. The Nafion-coated electrode achieved a DNA elution efficiency of over 70%, compared to less than 10% for uncoated gold. The retrieved DNA remained intact and was successfully amplified via polymerase chain reaction (PCR), highlighting Nafion’s critical role in preserving DNA integrity and enabling reusable biosensing platforms.

PDMS is extensively used in microfluidic device fabrication, but integrating ion-exchange membranes like Nafion has been technically challenging due to the lack of direct bonding between Nafion and cross-linked PDMS. To overcome this, a study suggests a simple and effective method for achieving direct integration of Nafion membranes into PDMS structures using a bifunctional silane crosslinker [51]. By applying plasma treatment to both Nafion and PDMS, reactive surface groups are generated that covalently bond with the silane, resulting in strong, stable and uniform adhesion at the interface. Peel tests confirm the robustness of the bond, which remains stable even in acidic environments and the ionic conductivity of the integrated Nafion remains comparable to commercial standards, making this approach highly viable for electrochemical microfluidic applications.

### 2.6. Implants

A Nafion-based modification strategy was proposed for enhancing the long-term performance and biocompatibility of implantable biomedical devices. In particular, the Nafion surface was chemically modified with a copolymer blend of 10% 2-hydroxyethyl methacrylate (HEMA) and 90% tetraglyme to create a coating that is not prone to nonspecific protein adsorption. Once this antifouling base was established, the surface was further functionalized by covalently bonding the bioactive tyrosine-arginine-glycyl-aspartic acid-serine YRGDS peptide, which is known to promote integrin-mediated cell adhesion [52]. Experimental evidence indicated reduced production of type I collagen (that is known to be the main component of a fibrotic capsule that eventually covers an implant) and more favorable normal human dermal fibroblasts (NHDFs) cell viability and spreading on the peptide-decorated, non-fouling Nafion.

Nickel-titanium (Ni-Ti) orthodontic wires are favored in dental applications due to their superior mechanical properties and affordability, but their susceptibility to oral corrosion presents challenges with respect to their durability, esthetics and biocompatibility. A study investigated the enhancement of corrosion resistance and lifespan of Ni-Ti wires via electrochemical deposition of ZrO_2_ followed by Nafion coating. A notable reduction in the corrosion rate from 4.436 × 10^−1^ mm/year in uncoated wires to 4.068 × 10^−1^ mm/year in Nafion/ZrO_2_-coated wires was observed [53].

Another study identified a short peptide (WIWHCW) that binds selectively and reversibly to Nafion, with a dissociation constant of approximately 140 µM [54]. Its histidine and tryptophan residues form the key binding motifs, enabling stable yet tunable interactions. This study paves the way for a versatile bio-anchoring strategy for immobilizing enzymes, antibodies, or cell-adhesive molecules on Nafion-based biosensors, lab-on-a-chip devices and implant coatings.

### 2.7. Biofluid Profiling

Nafion has been effectively integrated into ambient ionization mass spectrometry (MS) platforms, providing a powerful tool for the in situ desalting and direct analysis of biological fluids [55]. The approach relies on the cation-exchange capacity of Nafion applied on the surface of spray tips to remove interfering inorganic salts such as Na^+^ and K^+^, thereby enhancing ionization efficiency and simplifying MS data. This approach is particularly important for biological sample analysis, where high salt content often leads to ion suppression and the formation of multiple adducted ion species, complicating both qualitative and quantitative analyses. With demonstrated desalting efficiency reaching up to 90% and a salt tolerance of up to 100 μmol, this method significantly enhances the signal-to-noise ratio and enables more accurate detection of small-molecule drugs, metabolites and proteins.

### 2.8. Drug Delivery and Therapeutics

Thin films composed of Nafion and poly(allylamine hydrochloride) (PAH) were fabricated using a layer-by-layer (LbL) deposition technique and subsequently loaded with insulin by immersing the films in its aqueous solution [24]. Insulin release was enhanced at pH 2.0 due to protonation, which neutralizes its negative charge and weakens electrostatic interactions with the PAH layers. At pH 9.0, reduced attraction between insulin’s amino groups and the negatively charged PAH similarly promotes release. Notably, insulin release from these films was significantly enhanced at pH 2.0 and pH 9.0 compared to pH 7.4, demonstrating a strong pH-responsive behavior. This indicates that the Nafion/PAH LbL films not only provide effective protection for insulin but also offer a promising platform for controlled delivery, particularly in applications requiring targeted or stimulus-responsive drug release under mild conditions.

Nafion-based hollow capsules, constructed via sequential electrostatic deposition of Nafion and Fe^3+^ onto the surface of sacrificial polystyrene latex colloidal templates were shown to exhibit exceptional hydrolytic stability across a broad pH range and at elevated temperatures [56]. The capsules show excellent biocompatibility and chemical stability, while their permeability of small molecules such as fluorescein can be precisely tuned by altering the number of Nafion/Fe^3+^ bilayers.

**Figure 8:**
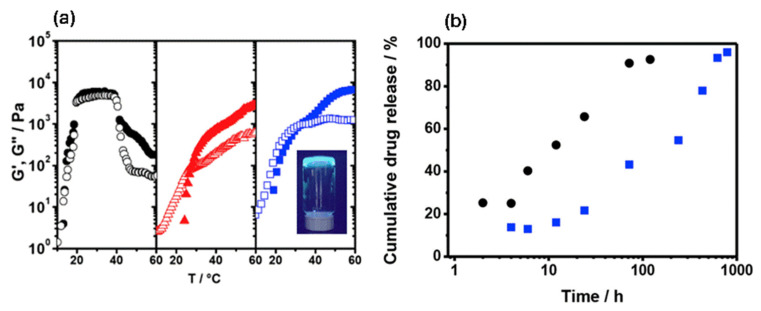
(**a**) Temperature sweeps (ω = 1 rad/s, strain amplitude = 2%) for aqueous gels containing 32.5 wt% E_19_P_69_E_19_ in the absence (●, black) and presence of 5 wt% Nafion (▲, red) and 10 wt% Nafion (■, blue). Solid symbols represent storage modulus (G′), and open symbols represent loss modulus (G″). Inset: Photograph of the 32.5 wt% E_19_P_69_E_19_/10 wt% Nafion gel at 40 °C under UV light (fluorescent dye added for visualization). (**b**) Release profiles of ibuprofen from 32.5 wt% E_19_P_69_E_19_ in the absence (●, black) and presence (■, blue) of 10 wt% Nafion in phosphate-buffer solution (pH 7.4) at 37 °C. Adopted from Reference [57].

A novel class of biocompatible hybrid hydrogels formed by combining Nafion with poly(ethylene oxide)-based block copolymers (namely E_19_P_69_E_19_, where E stands for ethylene oxide and P for propylene oxide) has been reported [57]. The hybrid systems maintain their injectable nature at room temperature but rapidly transform to robust hydrogels at physiological temperature (the rheological profiles of gels containing 32.5 wt% E_19_P_69_E_19_ in the absence and presence of 5 wt% Nafion and 10 wt% Nafion are shown in Figure 8a). At the same time, the hybrid systems facilitate sustained release of ibuprofen over a period of 26 days compared to just 3 days for the Nafion-free counterpart (Figure 8b). This sustained release makes the hybrid systems strong candidates for long-term therapeutic delivery, reducing the frequency of administration and improving patient compliance. In principle, Nafion-reinforced hydrogels can serve as injectable scaffolds for tissue regeneration and 3D-bioprinting bioinks that need both printability and mechanical strength.

A recent study disclosed Nafion-containing NPs capable of establishing ionic interactions with 1,3,5-triaza-7-phosphaadamantane (PTA), a protonable ligand used for Pt coordination in drug delivery systems [58]. Sulfonic acid groups of Nafion selectively protonate the nitrogen atoms of PTA, without interfering with the phosphorus atoms, thereby preserving PTA’s ability to coordinate Pt and allowing the formation of a stable Nafion NPs/PTAH^+^–Pt complexes. Nafion NPs particles were shown to be non-toxic to human cancer cell lines K562 (leukemia) and A2780 (ovarian carcinoma).

Ionic Polymer–Metal Composites (IPMCs), consisting of Nafion membranes coated with conductive metals, deform or bend under low-voltage electric fields due to the migration of hydrated cations and this biomimetic actuation response is ideal for integrated technologies such as medical implants and artificial muscles. Nafion-based actuators have been effectively employed in micropumps to deliver insulin precisely through hollow microneedles, providing a painless, adjustable and wearable method for insulin administration [59]. A major advantage of Nafion-IPMC systems is their ability to operate at very low voltages (3–6 V), making them safe for biomedical applications and well-suited for wearable or implantable devices.

Radiation therapy using beta particles offers a highly localized and effective treatment for superficial skin lesions such as melanoma or basal cell carcinoma. Yttrium-90 (Y-90), a pure beta emitter, is particularly well-suited for this application because of its favorable energy profile and short tissue range [60]. In clinical research, Y-90 has been incorporated into microspheres or immobilized within biocompatible Nafion membranes to create topical skin patches. These patches ensure uniform distribution of radioactivity and precise treatment targeting, offering a minimally invasive therapy.

### 2.9. Diagnostics and Imaging

**Figure 9:**
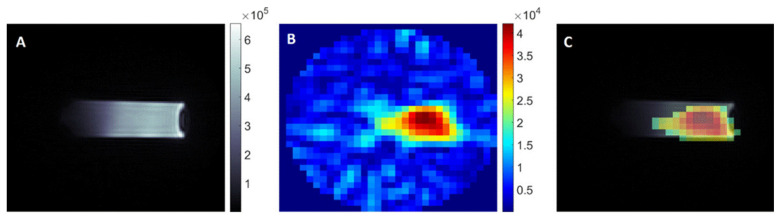
(**A**) ^1^H MR image of an agar phantom containing Nafion based nanocarriers. (**B**) Corresponding ^19^F MR image. (**C**) Overlay of the ^1^H and ^19^F MR images. Adopted from Reference [63].

Conventional Magnetic Resonance Imaging (MRI) agents based on paramagnetic Gd compounds that have been associated with nephrogenic systemic fibrosis (NSF) in patients with kidney problems [61], and possible brain deposition of Gd [62], thus necessitating the development of a new generation of Gd-free imaging agents. Nafion/poly-L-lysine (PLL) LbL nanocarriers were surface functionalized with pegylated poly-L-glutamic acid (PGA) and were explored as fluorine magnetic resonance imaging (^19^F MRI)-detectable drug delivery systems [63]. Figure 9A shows the ^1^H MR image of an agar phantom containing Nafion nanocarriers, Figure 9B displays the corresponding ^19^F MR image, while Figure 9C shows the overlay of the two images. A key advantage of this theragnostic approach lies in the near-zero natural abundance of ^19^F signal in the human body, allowing sufficient signal-to-noise ratio that facilitates the specific and unambiguous detection of exogenous fluorinated compounds. In doing so, these Nafion-based carriers can enable real-time imaging of tumor localization and drug accumulation.

### 2.10. Antimicrobial Surfaces

**Figure 10:**
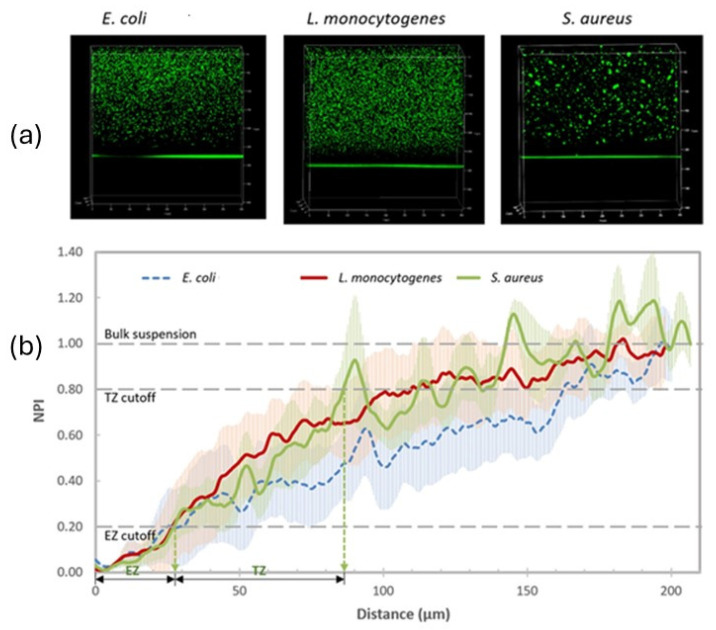
(**a**) 3D confocal laser scanning microscopy (CLSM) images showing the exclusion zone (EZ) at the Nafion–bacteria suspension interface for three pathogenic strains: *E. coli*, *L. monocytogenes*, and *S. aureus*. (**b**) Normalized pixel intensity (NPI) profiles indicate near-zero cell density within the first 25 μm from the Nafion–triptic soy broth (TSB) interface, a sharp increase between 25 and 50 μm, gradual decline after 80 μm, and a plateau beyond 150 μm. Adopted from reference [65].

It has been reported that Nafion-coated stainless steel disks showed significantly reduced adhesion of *Escherichia coli* (*E. coli*) [64] due to electrostatic repulsions between the negatively charged bacterial cells and the similarly charged coated surfaces. These findings suggest that the electrostatic properties of Nafion play a crucial role in mitigating bacterial adhesion, offering a promising approach to developing surfaces resistant to biofilm formation. Another study demonstrated that Nafion exhibits a bacterial exclusion zone (EZ) (Figure 10a) that can extend several micrometers from the surface and diminishes with time (Figure 10b), as van der Waals and acid-base forces start to dominate the Nafion-bacteria interactions [65]. This behavior has significant implications for developing antimicrobial surfaces, particularly in medical devices and food processing equipment, where biofilm formation poses a major challenge.

A study presents the development of a novel family of LbL coatings composed of Nafion, lysozyme and chitosan that demonstrate supreme resistance to microbial colonization, showing more than 99.99% growth inhibition of *E. coli* and *Staphylococcus aureus* (*S. aureus)* [66]. The findings highlight that Nafion-based synergistic platforms can activate potent antimicrobial mechanisms that extend beyond EZ effects and underscore the crucial roles of chemical composition, surface topography and wettability in achieving exceptional antimicrobial efficacy.

**Figure 11:**
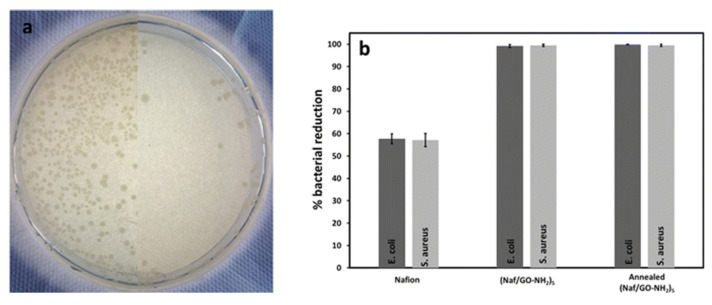
(**a**) Photographs of Petri dishes containing *E. coli* cultures. The left side shows exposure to uncoated crystals, while the right side shows exposure to (Naf/GO–NH_2_)_5_-coated disks under identical conditions. (Images are composites of two separate Petri dishes.) (**b**) Percent reduction in *E. coli* and *S. aureus* populations after exposure to Nafion, (Naf/GO–NH_2_)_5_, and thermally annealed-(Naf/GO–NH_2_)_5_ coated crystals. Adopted from Reference [67].

Recent studies have introduced durable, drug-free antimicrobial coatings based on negatively charged Nafion (ζ = −54.8 mV at pH 2.7) and positively charged amine-terminated graphene oxide (GO–NH_2_) (ζ = +26.7 mV at pH 2) [67]. This alternating multilayer structure is chemically stable and mechanically resilient and resists detachment even under strong friction forces. Impressively, the Nafion/GO–NH_2_ coating exhibits antimicrobial activity, achieving over 99% inhibition of both *E. coli* and *S. aureus* and maintains its performance after thermal annealing at 200 °C (Figure 11a,b). Those performance characteristics make it highly attractive for medical devices, hospital surfaces and food processing equipment, where long-term antimicrobial action and the ability to withstand dry sterilization conditions are essential.

A follow-up study introduced a similar waterborne LbL nanocoating, this time combining Nafion with positively charged imidazole-functionalized graphene quantum dots (GQD-Ims) [68]. This nanocoating inhibited the growth of both Gram-positive and Gram-negative bacteria by more than 99% and retained its functionality even after prolonged exposure to 200 °C, thus meeting requirements for dry heat sterilization. Moreover, the Nafion/GQD-Im coating demonstrated UV shielding properties, effectively protecting sensitive materials from ultraviolet-induced degradation, as validated by dye decomposition experiments. The combination of thermal stability, chemical resistance, antibacterial efficiency and UV protection highlights the potential of Nafion-based LbL nanocoatings in advanced packaging systems, cosmetics, pharmaceutical preservation and disinfection of reusable medical tools.

## 3. Outlook

Nafion’s unique combination of high proton conductivity, chemical stability and biocompatibility has positioned it as a cornerstone material for the development of autonomous biomedical devices. These self-powered systems are designed to operate independently within the body, leveraging physiological conditions such as ionic gradients or glucose levels to generate electrical energy [25]. Furthermore, the electrochemical responsiveness of Nafion also enables dynamic sensing and actuation functions, making it a valuable component in closed-loop systems that adapt to the patient’s biological state. For example, a cutting-edge Nafion-based micropump was specifically engineered for painless and tunable insulin delivery using microneedles, paving the way to a commercial, user-friendly diabetic care device [26].

Medical-grade Nafion tubes (commercialized by Perma) are widely used in respiratory and diagnostic medical devices due to their exceptional ion exchange properties and high moisture transfer rates. These tubes are designed to selectively allow water vapor transport while blocking contaminants, making them ideal for applications such as capnography, anesthesia monitoring and therapeutic gas humidification.

Experimental evidence suggests that Nafion can enhance the structural integrity, thermal responsiveness and biostability of soft biomaterials, which is vital for extended therapeutic action. Nafion-reinforced hydrogels, with improved mechanical strength, are well-suited for injectable scaffolds in 3D bioprinting and tissue engineering and load-bearing implants with localized drug delivery, minimizing systemic side effects [57]. Similarly, CNT/Nafion composites show promise for nerve repair scaffolds and electro-stimulative therapies [45]. In nanomedicine, Nafion-based ^19^F MRI-visible nanocarriers can provide a multifunctional platform for targeted insulin, non-invasive imaging and real-time disease monitoring [63].

Electrostatically assembled Nafion-based nanocoatings combined with functionalized graphene and carbon-based NPs have demonstrated exceptional efficacy in eliminating pathogens such as *E. coli* and *S. aureus*, even after exposure to high temperatures and harsh chemicals [67,68]. This drug-free antimicrobial strategy is particularly appealing for long-term use in medical devices, surgical tools and hospital surfaces.

Despite Nafion’s significant promise in biomedical applications, several critical challenges must be addressed before widespread clinical adoption can be realized. One of the major concerns lies in ensuring the long-term biostability of Nafion composites in vivo. While Nafion exhibits excellent chemical and thermal stability, the complex and dynamic environment within the human body, marked by enzymatic activity, pH fluctuations and immune surveillance, demands a deeper understanding of how this polymer performs over time. Furthermore, translating laboratory-scale prototypes into clinical-grade materials necessitates strict control over Nafion’s synthesis, purification and fabrication processes to ensure batch-to-batch consistency and regulatory compliance.

A notable contribution provides a comprehensive evaluation of the toxicity of Nafion and its combustion products in mice following a 24-day exposure, revealing multiple mechanisms of hepatotoxicity [69]. Histopathological analyses showed hepatocellular necrosis and inflammatory infiltration, particularly linked to fluoride (F^−^) and organofluorine byproducts generated under oxygen-limiting combustion. Biomarker assays indicated oxidative stress via suppression of antioxidant enzymes, though no lipid peroxidation was detected. Transcriptomic profiling further revealed disruption of xenobiotic metabolism (e.g., cytochrome P450s and UGTs), carbohydrate and lipid metabolic pathways and key biological processes, including signal transduction, cellular processes and immune responses. These findings suggest that both direct ingestion and combustion-derived exposure to Nafion may trigger liver toxicity through oxidative stress, immune activation and metabolic dysregulation.

Another fundamental limitation is Nafion’s non-biodegradable nature, which poses a significant challenge for applications involving temporary implants or bioresorbable devices. Its persistence in the body may require secondary surgical procedures for removal, thereby increasing patient risk and overall healthcare costs. Additionally, concerns arise about the long-term fate of residual fragments or byproducts resulting from potential mechanical degradation or wear.

Nafion is well-regarded for its excellent proton conductivity and outstanding chemical stability, which make it highly suitable for bioelectronic devices that rely on efficient ionic conduction. In contrast, chitosan and polyethylene glycol (PEG) offer better biodegradability, while sulfonated polyether ether ketone (SPEEK) is suitable for load-bearing biomedical applications, and sulfonated polyimides maintain strength and flexibility, greater tunability and more cost-effective production suitable for implantable devices.

From an environmental perspective, the presence of persistent byproducts such as Nafion BP2, a member of the per- and polyfluoroalkyl substances (PFAS) family, poses ecological and public health concerns [70,71]. This compound has been detected in industrial effluents, wildlife and human biological samples, underscoring the need for systematic studies on its environmental persistence and potential toxicological impact [72,73]. In a 7-day oral exposure study, male and female Balb/c mice showed hepatic steatosis and activation of liver-associated transcription factors, including constitutive androstane receptor, pregnane X receptor and nuclear factor erythroid 2-related factor 2, but not peroxisome proliferator-activated receptor α [74]. Benchmark dose analysis revealed transcriptional effects at doses as low as 0.04 mg/kg/day, with xenobiotic metabolism and cell cycle pathways being most sensitive. These findings suggest potential hepatotoxicity and point to the need for the establishment of exposure guidelines for Nafion BP2.

## 4. Future Perspectives

Moving forward, further research efforts are needed to advance the seamless integration of Nafion into biological tissues and living systems, with the aim of maximizing its impact in precision medicine. One of the most promising avenues involves the incorporation of targeting ligands, imaging agents and nanomaterials (such as CNTs, graphene derivatives, metallic NPs and quantum dots) into Nafion matrices. These hybrid systems can significantly expand Nafion’s capabilities by improving its proton conductivity, mechanical strength and responsiveness to environmental stimuli.

In addition to its expanding role in biosensing and drug delivery, future developments may involve Nafion applied for the build-up of bioelectronic interfaces that can monitor and modulate physiological signals in real-time. Nafion’s ionic conductivity and flexibility make it a promising candidate for the development of soft, implantable devices for neural interfacing or cardiac monitoring. Furthermore, its chemical stability and biocompatibility can be exploited to develop scaffolds for tissue engineering, where it could provide structural support and facilitate localized cell growth and differentiation.

Integration with next-generation wearable and implantable medical devices is also emerging, given that Nafion could play a pivotal role in autonomous energy harvesting systems, function as a smart membrane for continuous biofluid analysis (such as sweat, saliva, and blood), or serve as a structural template for stretchable, conductive fibers in flexible electronics.

However, broader clinical adoption will also depend on addressing key challenges related to cost and scalability. Currently, Nafion remains relatively expensive due to its complex manufacturing process and reliance on fluorinated precursors. For large-scale biomedical application, cost-effective synthesis and improved production efficiency remain to be established. Moreover, scaling up production without compromising material consistency, biocompatibility, or performance represents a major challenge toward making Nafion-based technologies commercially viable and accessible worldwide.

## 5. Conclusions

Initially established as the gold standard in fuel cell technology, Nafion’s role has significantly evolved, with its utility now extending far beyond energy systems. In recent years, Nafion has played an increasingly important role in the development of high-performance sensors capable of real-time monitoring in complex physiological settings, implantable biomedical devices, smart drug delivery platforms and antimicrobial coatings. These advances are largely driven by its unique combination of properties (proton conductivity, chemical and thermal stability, biocompatibility, and selective ion permeability) which collectively make it exceptionally well-suited for interfacing with both biological systems and electrochemical environments. The integration of functional NPs into Nafion is expected to enable synergistic effects, driving further advances in smart surfaces, precision medicine and targeted therapies. Despite Nafion’s great promise in biomedicine, key challenges include its non-biodegradable nature, the demand for long-term biostability in vivo and the necessity of strict manufacturing controls to ensure safety and regulatory compliance for clinical use. Additionally, environmental concerns associated with byproducts like Nafion BP2 highlight the need for further toxicological and ecological studies.
